# Exosomes Derived from RM-1 Cells Promote the Recruitment of MDSCs into Tumor Microenvironment by Upregulating CXCR4 via TLR2/NF-*κ*B Pathway

**DOI:** 10.1155/2021/5584406

**Published:** 2021-10-08

**Authors:** Nan Li, Yingying Wang, Haoyu Xu, Hexi Wang, Yingying Gao, Yao Zhang

**Affiliations:** ^1^Department of Urology, The First Affiliated Hospital of Chongqing Medical University, Chongqing 402160, China; ^2^Chongqing University Cancer Hospital, Chongqing 400030, China; ^3^Department of Oncology, People Hospital of Jiulongpo District, Chongqing 400050, China; ^4^Department of Laboratory Diagnosis, People's Hospital of Banan District, Chongqing 401300, China

## Abstract

Myeloid-derived suppressor cells (MDSCs) play a critical role in tumor immune escape because of its remarkable immunosuppressive effect. However, the mechanism of MDSCs migrated into tumor microenvironment remains unclear. In this study, we demonstrated the recruitment of MDSCs can be promoted by exosomes derived from prostate cancer cells, which could upregulate chemokine (CXC motif) receptor 4 (CXCR4) via the TLR2/NF-*κ*B signalling pathway. Flow cytometry detected that the percentage of MDSCs in the mice spleen and tumor tissue was significantly increased after injection with exosomes via mouse tail vein. Transwell chemotaxis assay showed the recruitment of MDSCs toward the lower chamber was enhanced after stimulation with exosomes, and the migration ability could be inhibited by AMD3100 (a CXCR4 specific inhibitor) both in vivo and in vitro. Additionally, Western blot and flow cytometry verified a remarkably increase of CXCR4 in MDSCs after incubation with exosomes; meanwhile, the protein level of TLR2 and activation of NF-*κ*B were also strengthened obviously. Nevertheless, after blocking TLR2 by C29 (a TLR2-specific inhibitor), the expression of p-p65 and CXCR4, which were hypothesized as the downstream target of TLR2, was prominently reduced. In conclusion, prostate cancer-derived exosomes could reinforce CXCR4 expression in MDSCs through the TLR2/NF-*κ*B signalling pathway, eventually promoting migration of MDSCs into tumor microenvironment in a CXCR4-CXCL12 axis-dependent manner.

## 1. Introduction

As one of the most prevalent cancers worldwide [1], prostate cancer (PCa) in the early stage does not emerge obvious symptoms, consequently evolving into castration-resistant PCa (CRPC) along with local or distant metastasis [2]. Many studies have shown that tumor-associated immune cells are important in the development of prostate cancer [3, 4]; however, the mechanisms by which immunosuppression cells migrated to tumor microenvironment are still unclear.

As a microparticle immune carrier, exosomes have been found to be involved in distant organs and are important in antitumor immunity. They can transmit bioactive components from plenty of cells such as cancer cells, immune cells, and fibroblast cells, as well as mesenchymal cells [5] into the recipient cells. These diameters among 40–100 nm vesicles have been widely reported in promoting cancer development in respects of cell proliferation, migration, metastasis, angiogenesis, and chemoresistance [5, 6]. In view of their distinguished efficacy in various cancer, they were also considered as vehicles of potential biomarkers in cancer diagnosis [7, 8]. Recently, how exosomes are released by cancer cells in the tumor microenvironment and uptaken by different immune cell types, thereby, accelerating immune evasion and affecting tumor development [9] is probed in depth.

Myeloid-derived suppressor cells (MDSCs) contained highly heterogeneous cells derived from bone marrow immature myeloid progenitors. They are generally identified to be Gr-1^+^CD11b^+^ in mice and CD11b^+^CD33^+^ in human. In normal mice, MDSCs range usually 20–30% in the bone marrow (BM) and 2–4% in the spleen [10]. Two main types of MDSCs have been identified as granulocytic (G-MDSC) represented by CD11b^+^Gr-1^hi^Ly-6G^+^Ly-6C^low^ and monocytic (M-MDSC) represented by CD11b^+^Gr-1^hi^Ly-6G^−^Ly-6C^hi^ [11–13]. In many studies, MDSCs have different immunosuppressive pathways to facilitate tumor development such as suppressing effector T (Teff cells) cells and inducing regulatory T (Treg) cells or regulatory B cells under certain conditions [10, 12], and some tumor-derived factors such as CSF-1, IL-6, IL-10, VEGF, and GM-CSF could influence recruitment of the MSCs at tumor site [14]. Another study has shown that T cell dysfunction could be inducted by MDSC through producing TGF-*β*, ROS, L-arginine metabolism, and peroxynitrites [15]. Moreover, MDSCs contribute to tumor development and vascularization through enhancing MMP9 and differentiating into tumor endothelial cells [16].

CXC motif chemokine ligand 12 (CXCL12) is a chemokine produced constitutively by various types of tumor cells and stromal cells [17, 18]. CXCL12/CXCR4 inflammatory signalling may affect the efficient chemotaxis function of inflammatory cells such as neutrophils, lymphocytes, and monocytes [19, 20]. Besides, it also accumulated immunosuppression cells such as Treg cells, dendritic cells, and MDSCs to tumor microenvironment to exert their immunosuppression and finally promote tumor development [21–23].

The present study aimed at investigating the importance of exosomes in upregulating CXCR4 expression to influence the migration of MDSCs and confirming the underlying mechanism. We observed a reinforcement of phosphorylation NF-*κ*B and TLR2 in exosome-incubated MDSCs. Notably, we demonstrated a latent mechanism that exosomes could heighten the activation of the TLR2/NF-κB signalling pathway, resulting in the augment of CXCR4 expression and gathering of MDSCs into tumor microenvironment.

## 2. Materials and Methods

### 2.1. Cell Culture

Cell line of mouse PCa, RM-1, was bought from the Cell Bank of Chinese Academy of Sciences, and the cells were cultured in RMPI 1640 medium (Gibco; Thermo Fisher Scientific, Inc. Waltham, MA, USA) containing 10% FBS (Gibco; Thermo Fisher Scientific, Inc.), 100 U/ml penicillin, and 100 mg/ml streptomycin (Gibco; Thermo Fisher Scientific, Inc.) at 37°C in a 5% CO_2_ condition.

### 2.2. Exosomes Isolation

The supernatant of RM-1 cells was collected after culture with exosomes-free FBS (the bovine exosomes were depleted through centrifugation at 100,000 × g at 4°C for 2 h) for 48 h. The media was centrifuged at 300 × g for 10 min, 2,000 × g for 20 min, and 10,000 × g for 30 min (Ultracentrifuge Thermo Electron LED GmbH, Germany). 100 KDa molecular weight cutoff (MWCO) centrifugal filter (EMD Millipore, Billerica, MA, USA) was used to enrich exosomes by ultrafiltration at 1,000 × g for 30 mins; then, preenrichment of exosomes media was ultracentrifuged at 100,000 × g for 90 min. (Ultracentrifuge CP100WX; Hitachi Koki Himac, NuAire, Tokyo, Japan). After resuspension, ultracentrifugation, and resuspension, exosome solution passed through a 0.22 *μ*m filter and stored at −80°C. The exosome concentration was measured with the BCA protein assay kit (Beyotime Institute of Biotechnology, Shanghai, China).

### 2.3. Electron Microscopy and Biomarkers Analysis

Suspension of exosomes pellet was mounted onto cooper grids coated with carbon through floating the droplet on the grid for 5 min. Excessive liquid was removed with a piece of dry filter paper. Then, the cooper grid was washed 3 times in PBS and stained with 2% phosphotungstic acid (Servicebio, China) for 2 min. Samples were visualized under a HT7700 electron microscopy (Hitachi, Japan) to observe the form of exosomes. Biomarkers of exosomes included HSP70, TSG101, and CD63 that were examined by Western blot. 20 *µ*g of exosomes was fractionated by 10%–15% SDS-PAGE and transferred to PVDF membranes (Millipore). The membranes were blocked with 5% skim milk in TBS-T buffer for 1 h at room temperature. Rabbit anti-mouse monoclonal antibodies of HSP70, TSG101, and CD63 (Abcam, USA) were diluted to 1 : 1000 and incubated at 4°C overnight. The blots were incubated with the appropriate horseradish peroxidase- (HRP) tagged goat anti-rabbit secondary antibodies (Abcam, USA) (diluted with the ratio of 1 : 5,000) for 30 min, and the labeled proteins were detected by an Odyssey Infrared System (LI-COR Bioscience, Lincoln, NE, USA).

### 2.4. MDSCs Isolation

Erythrocyte-free single cell suspensions were obtained by grinding spleens on a 200 mesh strainer and incubated with red blood cell lysis buffer (Beyotime Institute of Biotechnology) at 4°C for 5 min, according to Xu et al. [24]. Cells were incubated with magnetic beads (Miltenyi Biotec, Germany) and then loaded onto a magnetic column to gain CD11b^+^Gr-1^+^ MDSCs (including Gr-1^+^Ly-6G^+^ and Gr-1^+^Ly-6G^−^ MDSCs), following the instruction of the manufacturer [S1].

### 2.5. Transwell Chemotaxis Assay

MDSCs were divided into four treatment groups by coculturing with PBS, exosomes, exosomes plus AMD 3100 (MedChemExpress, USA) of 10 *μ*g/500 *μ*l, and AMD 3100 for 24 h, respectively. Then, MDSCs were harvested, adjusted to a concentration of 5 × 10^5^/200 *μ*l RPMI 1640, added into the upper chambers, and divided into four different treatment groups: PBS, exosomes, exosomes plus AMD3100, and AMD3100. CXCL12 was added in the lower chamber and cocultured for 24 h. 500 *μ*l RPMI 1640 with 100 ng CXCL12 (Abcam, USA) was added into the lower chambers and then incubated at 37°C for 24 h, according to Liu et al. [25]. The cells migrated to the lower surface had been fixed with 4% paraformaldehyde for 15 min and stained with 0.1% crystal violet for 10 min. Cells were counted and averaged through the selection of 5 random fields/well under a light microscope (Nikon, Japan).

### 2.6. Tumor Model

2 × 10^6^ RM-1 cells were resuspended in 200 *μ*l PBS and injected subcutaneously in the right/left back of male C57/B6 mice (6–8 weeks). Tumor size was detected by the caliper every two days and calculated based on formula *V*=*π*/6 × *L* × *W*^2^ (*L* = length; *W* = width). Two weeks later, the mice were divided into four groups (6 mice in each group), which were treated with PBS, exosomes, AMD 3100, and AMD 3100 + exosomes, respectively. Then, mice were injected via tail vein three times per week for one week. One day after the final injection, overdose inhalation anesthesia (2-3% isoflurane) was used before euthanasia with carbon dioxide (CO_2_); then, tumor tissues were isolated for further analysis. The maximum diameter of the tumor did not exceed 2 cm. This study was approved by the Ethics Committee of Chongqing Medical University, Chongqing, China.

### 2.7. Flow Cytometry

Erythrocyte-free single cell suspensions from the spleen and tumor were incubated with anti-CD11b-APC (Biolegend, USA) and anti-Gr-1-PE (Biolegend, USA) antibodies at 4°C for 15 min in dark. After washed in buffer and centrifuged, the cell precipitation was resuspended. The percentage of CD11b^+^ Gr-1^+^ double-positive MDSCs was analyzed by flow cytometry (Beckman Coulter, Pasadena, CA, USA). To determine the CXCR4-positive expression rate in the CD11b^+^ Gr-1^+^ MDSCs group, normal mice were divided into two groups, which were treated with exosomes and PBS, respectively. The single cell suspension from the bone marrow and spleen of two groups were stained by anti-CD11b-FITC (Biolegend, USA), anti-Gr-1-PE (Biolegend, USA), and anti-CD184-APC (Biolegend, USA) antibodies and then analyzed by flow cytometry.

### 2.8. Cocultured and Western Blot

In order to verify whether exosomes could increase the expression of CXCR4 on the surface of MDSCs through the TLR2/NF-κB signalling pathway, MDSCs were incubated with exosomes (20 *μ*g/ml) or PBS, with or without TLR2-specific inhibitor C29 (50 *μ*M, HY-100461, MedChemExpress, USA), cultured in RPMI 1640 with GM-SF (10 ng/ml, Peprotech EC, USA) for 24 h, and then, the cells were obtained. The expression levels of CXCR4, p65, p-p65, and TLR2 in MDSCs were detected by Western blot as described before. The antibodies CXCR4, p65, p-p65, and TLR2 were purchased from Abcam, USA.

### 2.9. Statistics

All the experiments had been repeated for 3 times, and these data were analyzed by GraphPad 10. Student's *t*-test was carried out for comparison between two groups, and comparison in three or more groups was conducted by one-way analysis of variance (ANOVA) followed by Tukey's multiple comparison tests. *P* < 0.05 was considered as significant.

## 3. Results

### 3.1. Identification of Exosomes

**Figure 1:**
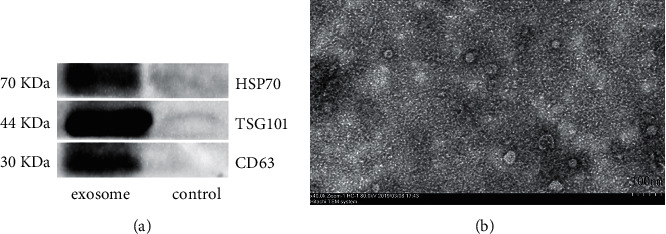
Identification of exosomes derived from RM-1. (a) Biomarkers CD63, HSP70, and TSG101 expression in exosomes detected by Western blot. Exosomes-depleted supernatant was set as the control group. (b) Purified exosomes observed by electron microscopy after ultrafiltration centrifugation; result showed exosomal pallet-like lipid bilayer vesicles and the average diameter was among 30–80 nm. The scale bar is 100 nm.

Exosomes were isolated and purified from the supernatants of RM-1 cells by ultrafiltration centrifugation. Western blot showed the expressions of CD63, HSP70, and TSG101 were higher in the exosomes group than the exosomes-depleted supernatants set as the control group (Figure 1(a)). In addition, electron microscopy was applied on observing the pallet-like structure of lipid bilayer vesicles with the similar size, and the average diameter was among 30–80 nm (Figure 1(b)).

### 3.2. The Percentages of MDSCs in the Spleen and Tumor Tissue Were Increased in Tumor-Bearing Mice with the Time

**Figure 2:**
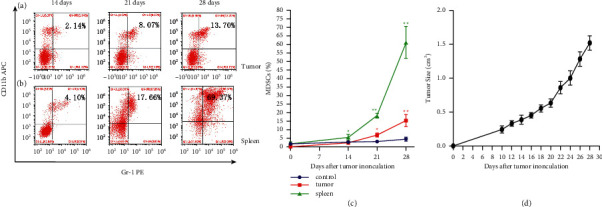
The percentage of MDSCs in the spleen and tumor tissue increased with time after subcutaneous injection in mice. Mice were executed at the time points of 14 days, 21 days, and 28 days after inoculated with RM-1 cells; the tumor tissue ((a), (c)) and spleen ((b), (c)) were dissociated and prepared as single cell suspensions, respectively; then, investigate the percentage of MDSCs in each group by flow cytometry. Tumor size was detected by the caliper every two days, and the tumor growth curve of mice bearing RM-1 tumors is shown (d). Percentage of MDSCs in the normal mice spleen as the control group. ^*∗*^*P* < 0.05 and ^*∗∗*^*P* < 0.01 compared with the 14 days group.

The tumor was formed subcutaneously 10 days after injected; tumor size was tested by calipers every two days. Then, mice were executed at the time points of 14 days, 21 days, and 28 days, respectively. The percentage of MDSCs in the spleen and tumor tissue was investigated by flow cytometry. The results showed that with the prolongation of tumor-bearing time, the percentage of MDSCs accumulated into the tumor tissue (Figures 2(a) and 2(c)) and spleen (Figures 2(b) and 2(c)) increased following the tumor growth, and the tumor growth curve has shown the positive correlation with the increasing trend of MDSCs (Figures 2(d)). Tumor size was detected by the caliper every two days and calculated based on formula *V*=*π*/6 × *L* × *W*^2^ (*L* = length; *W* = width). Percentage of MDSCs in normal mouse spleens was set as the control group. This phenomenon might suggest that the migration of MDSCs into periphery and tumor microenvironment plays an important role in the development of tumor.

### 3.3. Exosomes Could Induce MDSCs Expansion and Migration, and the Recruitment Could be Inhibited by CXCR4 Inhibitor AMD3100 Both In Vivo and In Vitro

**Figure 3:**
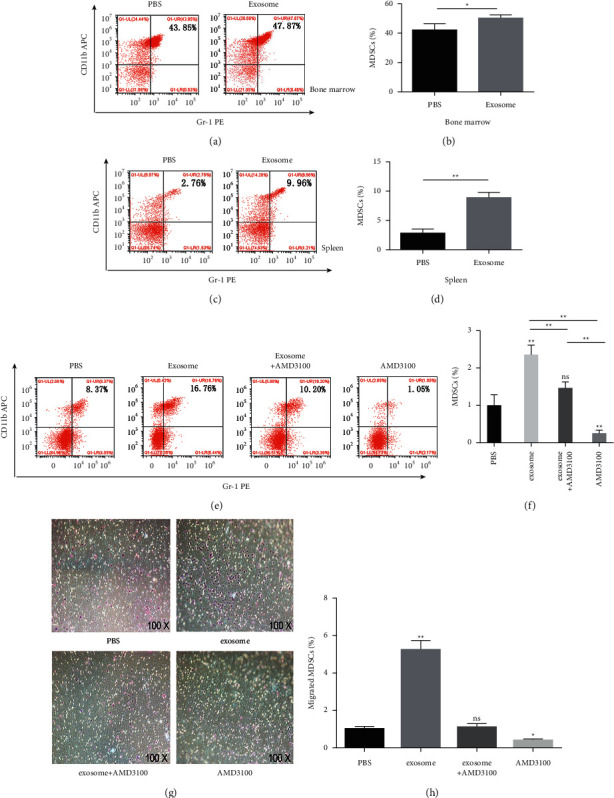
Exosomes induced MDSCs expansion and migration. The recruitment could be inhibited by AMD3100 both in vivo and in vitro. After injecting exosomes to normal mice via the tail vein 3 times per week for 2 weeks, the percentage of MDSCs in the BM ((a), (b)) and spleen ((c), (d)) was examined by flow cytometry. ((e), (f)) The percentage of MDSCs in tumor tissue of PBS, exosomes, exosomes plus AMD3100, and AMD3100 treatment groups measured by flow cytometry, respectively. ((g), (h)) In transwell chemotaxis assay, MDSCs from BM of mice bearing tumors were divided into PBS, exosomes, exosomes plus AMD3100, and AMD3100 treatment groups in the upper chamber; after cocultured with CXCL12 for 24 h, cells were counted and averaged through the selection of 5 random fields/well under a light microscope to detect the chemotaxis effect of CXCL12 to MDSCs. ^*∗*^*P* < 0.05 and ^*∗∗*^*P* < 0.01 compared with the PBS group.

After injecting exosomes to normal mice via the tail vein 3 times per week for 2 weeks, the bone marrow cells and splenocytes were obtained one day after the final injection. Flow cytometry was applied on detecting the proportion of CD11b^+^ Gr-1^+^ MDSCs. The result showed the proportion of MDSCs in BM (Figures 3(a) and 3(b)) and spleen (Figures 3(c) and 3(d)) was stronger, increased in the exosome group than the PBS group.

In tumor-bearing mice models, compared with the PBS group, the exosome group showed obvious recruitment of MDSCs in tumor site. Moreover, after injected with or without AMD3100, the group of exosomes plus AMD3100 showed the percentage of MDSCs significantly decreased more than the only exosomes group (Figures 3(e) and 3(f)). It might suggest that the accumulation effect of MDSCs induced by exosomes could be blocked by AMD3100 in vivo.

Transwell chemotaxis assay was executed to study whether the chemotaxis effects of CXCL12 on exosome-stimulated MDSCs can be enhanced in vitro. The results showed that the exosomes group could accumulate more MDSCs into the lower chamber compared with the PBS group, and exosomes plus the AMD3100 group showed the decreased migration effect of MDSCs than the only exosomes group (Figures 3(g) and 3(h)), which was consistent with the in vivo experiment.

Taken together, these results indicated that accumulation of MDSCs could be induced by exosomes, and after blocking the interaction of CXCL12 and CXCR4, the migration effect of MDSCs induced by exosomes could be suppressed by AMD3100 both in vivo and in vitro. It was believed that exosomes can influence the recruitment of MDSCs, which interfered by CXCR4.

### 3.4. Exosomes Upregulated the CXCR4 Expression of MDSCs Both In Vivo and In Vitro, Which Mediated by TLR2/NF-*κ*B Signal Pathway

**Figure 4:**
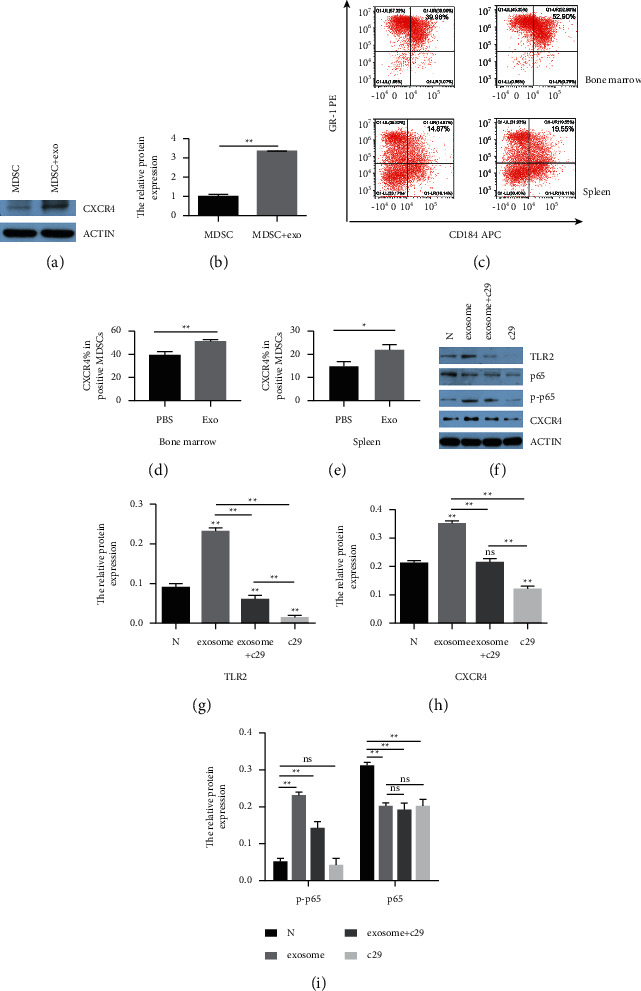
CXCR4 expression was increased in MDSCs treated with exosomes both in vivo and vitro, which mediated by activating NF-*κ*B and engaging TLR2. (a), (b) MDSCs cocultured with exosomes for 24 h and the expression of CXCR4 in MDSCs detected by Western blot. Exosomes and PBS 20 ug were injected into normal mice via the tail vein 3 times per week for 2 weeks, respectively; single cell suspensions from bone marrow ((c), (d)) and spleen ((c), (e)) were prepared one day after the final injection and then stained by anti-CD11b-FITC, anti-Gr-1-PE, and anti-CD184-APC antibodies to detect CXCR4 positive expression rate in population of CD11b + Gr-1 + MDSCs by flow cytometry. MDSCs were cocultured with PBS, exosomes, C29 plus exosomes, and C29 for 24 h; the expression levels of TLR2 ((f), (g)), p65, p-p65 (i), and CXCR4 (h) in each group were measured by Western blot. *β*-Actin served as the control. ^*∗*^*P* < 0.05 and ^*∗∗*^*P* < 0.01 compared with the control group.

To confirm whether exosomes could upregulate CXCR4 expression in MDSCs in vitro, MDSCs were cocultured with exosomes or PBS for 24 h. Western blot showed that MDSCs cocultured with exosomes expressed higher CXCR4 than the PBS group (Figures 4(a) and 4(b)). Next, exosomes were injected into mice tail vein. Flow cytometry showed that the expression of CXCR4 in the exosomes group was significantly increased than that of the PBS group both in bone marrow (Figures 4(c) and 4(d)) and spleen (Figures 4(c) and 4(e)). These data demonstrated that exosomes could upregulate the CXCR4 expression in MDSCs both in vivo and in vitro.

Subsequently, after cocultured with exosomes for 24 h, MDSCs were collected to detect the expression level of TLR2, phosphorylation of NF-*κ*B (p-p65), NF-κB (p65), and CXCR4. The Western blot showed that the engagement of TLR2 (Figures 4(f) and 4(g)) in MDSCs was significantly increased in the exosomes group compared with the PBS group and remarkably decreased by its specific inhibitor C29 (50 *μ*M) blocked for 24 h, while the expression tendency of p-p65 (Figure 4(i)) and CXCR4 (Figure 4(h)) exhibited similar results with TLR2. It confirmed that exosomes could act as an agonist of TLR2 and enhanced the expression of other two proteins; then, they were notably reduced right after the decreasing TLR2 blocked by C29, which might indicate that NF-*κ*B and CXCR4 are the downstream targets of TLR2 in the signalling pathway. Taken together, it was speculated that the upregulation of CXCR4 in MDSCs were mediated by exosomes through the TLR2/NF-*κ*B pathway.

## 4. Discussion

Recent studies have authenticated exosome as the carrier that includes a plenty of active biology competents which could achieve the crosstalk between tumor cells and immune cells. Exosomes play a dual role in cancer progression. On the one hand, they could modulate antigen presentation and immune activation and provide an effective antitumor immune response. For example, the exosomes enriched with Hsp70 can stimulate Th1-immune responses and elevate production of IFN-*α* and IgG2 in murine models [26]; exosomes with more HSP70 and MHC-I can induce DC maturation and strengthen the immune response [27]. On the other hand, exosomes have recently gained attention in promoting tumor development, especially in cancer immune surveillance and tumor escape responses. Exosomes facilitated angiogenesis [28], directly suppressed cytotoxic T lymphocytes and NK cells' antitumor responses, induced activation of immune suppressor cell subsets, and led to loss of tumor immune surveillance [29]. Moreover, another study reported that murine TS/A and 4T-1 breast tumor cell-derived exosomes induced MDSC morphology and activity in bone marrow myeloid cells (BMMCs), which caused tumor development and growth to increase concomitantly [30]. Consistent with these studies, our results indicated that treatment of mouse prostate cancer-derived exosomes noticeably enhanced the expansion of MDSCs in the bone marrow and recruitment to the spleen and tumor microenvironment.

Chemokine factors secreted from tumor and mesenchymal cells could form the concentration gradient to recruit white cells, and MDSCs are the important parts of them [31]. Recent research has shown that the CXCL12 expression level in prostate cancer was obviously higher than that in benign prostatic hyperplasia tissue [32]. Therefore, CXCL12 in the microenvironment of prostate cancer can recruit white blood cells expressing the corresponding receptor. In addition, CXCR4 is expressed extensively with high levels in all kinds of immune cells, including MDSCs, NK cells, and T cells [33]. Thus, it was supposed that, by affecting the CXCL12-CXCR4 chemotactic pathway, MDSCs could achieve the changes in migration capacity. Our data conformed to the previous work, indicating that compared with the PBS group, the exosome group showed significantly increased migration of MDSCs, and the MDSCs recruitment caused by exosomes could be suppressed by AMD3100 both in vivo and in vitro. In this article, it was demonstrated that exosomes upregulated CXCR4 expression in MDSCs, indicating the function of CXCL12-CXCR4 axis in promoting the attraction and retention of MDSCs into the tumor microenvironment. Additionally, the CXCL12-CXCR4 chemotactic pathway could also limit tumor growth through reducing tumor angiogenesis [34, 35].

Recent reports showed that HSP70 uses both TLR2 and TLR4 for transducing proinflammatory cytokine production through the MyD88/NF-κB signal pathway [36]. Our study also illustrated that Hsp70 was highly expressed in exosomes as biomarkers on its outer surface. TLR2 can recognize different types of deoxyadenosine, including HSP family proteins, and another study recognize that NK cells expressing TLR2 can receive the stimulation of exosomes in multiple myeloma [37] that also confirmed this view. Moreover, another finding indicated that Hsp72 located on the tumor exosomes surface can engage TLR2 expressed on MDSCs [38]. Our findings are similar with these results that exosomes coincubated with MDSCs induced TLR2 expression, indicating that the internalization of exosomes by MDSCs may be via the Hsp70 membrane-bound signal pathway.

Many previous research studies demonstrated that NF-*κ*B is one of the main pathways triggered by TLRs, and it is a transcription factor implicated in activation of several cytokine genes [39]. It was reported that genetic ablation of TLR2 in macrophages could abolish the NF-*κ*B activation effect stimulated by breast cancer-derived exosomes [40]. In our study, the evidence that exosomes which could enhance the activation of NF-*κ*B in MDSCs was also provided, whereas, preincubated with TLR2 inhibitor C29, the phosphorylation of NF-*κ*B (p-p65) were obviously decreased, and the expression of CXCR4 showed a similar trend with p-p65. Consistent with these findings, our experiment indicated that tumor derived exosomes could upregulate CXCR4 expression, putatively by increasing phosphorylation of NF-*κ*B, causing the expansion and migration of MDSCs.

**Figure 5:**
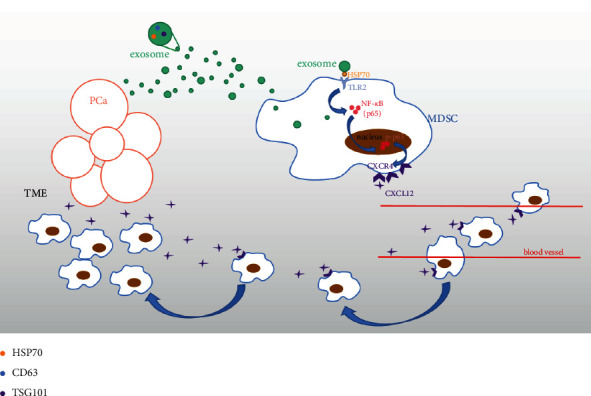
Schematic diagram of all the results.

In conclusion, new data were provided herein to support the hypothesis that exosomes secreted from prostate cancer could upregulate the expression of CXCR4 in MDSCs, putatively by increasing engagement of TLR2 and phosphorylation of NF-*κ*B, resulting in MDSCs accumulation into tumor microenvironment (Figure 5). In addition, the findings in this study also illustrated the possibility for CXCR4 to be a potential therapeutic target for treating prostate cancer, and inhibiting exosomal CXCR4 secretion may be a novel therapeutic against tumor development in the initial stage of prostate cancer.

Exosomes enriched with HSP70 were secreted from prostate cancer cells, inducing the engagement of TLR2 and phosphorylation of NF-*κ*B, resulting in upregulating expression of CXCR4 in MDSCs and eventually leading to the accumulation of MDSCs into tumor microenvironment (TME).

## Data Availability

The data used to support the findings of this study are included within the article.

## Supplementary Materials

Supplementary Materials Myeloid-derived suppressor cell isolation instruction.
