# Supplementary material for: Exosomes Derived from RM-1 Cells Promote the Recruitment of MDSCs into Tumor Microenvironment by Upregulating CXCR4 via TLR2/NF-κB Pathway
Source: J Oncol. 2021 Oct 8;2021:5584406. doi: 10.1155/2021/5584406 (PMC8519695; doi:10.1155/2021/5584406)
Supplement: Supplementary Materials — Myeloid-derived suppressor cell isolation instruction. [file 5584406.f1.pdf]

For further information refer to our website
www.miltenyibiotec.com

For technical questions, please contact your local subsidiary or distributor.

Technical Support Team, Germany:
Phone +49 2204 8306-830
macstec@miltenyi.com

Miltenyi Biotec

Miltenyi Biotec B.V. & Co. KG
Friedrich-Ebert-Straße 68, 51429 Bergisch Gladbach, Germany
Phone +49 2204 8306-0, Fax +49 2204 85197
macsde@miltenyi.com, **www.miltenyibiotec.com**

140-002-727.02

Miltenyi Biotec

Myeloid-Derived Suppressor Cell Isolation Kit
mouse

Order no. 130-094-538

Unless otherwise specifically indicated,
Miltenyi Biotec products and services are for research use only and not for diagnostic or therapeutic use.

Contents

1. Description
  1.1 Principle of the MACS® Separation
  1.2 Background information
  1.3 Applications
  1.4 Reagent and instrument requirements
2. Protocol
  2.1 Sample preparation
  2.2 Magnetic labeling of Gr-1^{high}Ly-6G^{+} cells
  2.3 Magnetic separation: Positive selection of Gr-1^{high}Ly-6G^{+} cells
  2.4 Magnetic labeling of Gr-1^{dim}Ly-6G^{-} cells
  2.5 Magnetic separation: Positive selection of Gr-1^{dim}Ly-6G^{-} cells
3. Example of a separation using the Myeloid-Derived Suppressor Cell Isolation Kit
4. References

1. Description

Components

2 mL Anti-Ly-6G-Biotin (MDSC-Kit), mouse: monoclonal antibodies conjugated to biotin (isotype: rat IgG2a).

2 mL Anti-Gr-1-Biotin (MDSC-Kit), mouse: monoclonal antibodies conjugated to biotin (isotype: rat IgG2b).

2×2 mL Anti-Biotin MicroBeads: MicroBeads conjugated to monoclonal anti-biotin antibody (isotype: mouse IgG1).

2 mL Streptavidin MicroBeads (MDSC-Kit): MicroBeads conjugated to streptavidin.

1 mL FcR Blocking Reagent, mouse

Capacity For 2×10^{9} total cells, up to 20 separations.

Product format All components are supplied in buffer containing stabilizer and 0.05% sodium azide.

Storage Store protected from light at 2–8 °C. Do not freeze. The expiration date is indicated on the vial label.

1.1 Principle of the MACS Separation

The Myeloid-Derived Suppressor Cell Isolation Kit has been developed for the isolation of Gr-1^{high}Ly-6G^{+} and/or Gr-1^{dim}Ly-6G^{-} myeloid cells. First, Gr-1^{high}Ly-6G^{+} cells are indirectly magnetically labeled with Anti-Ly-6G-Biotin (MDSC-Kit) and Anti-Biotin MicroBeads. Then,

the cell suspension is loaded onto a MACS® Column, which is placed in the magnetic field of a MACS Separator. The magnetically labeled Ly-6G+ cells are retained within the column. The unlabeled cells run through; this cell fraction is thus depleted of Gr-1highLy-6G+ and pre-enriched for Gr-1dimLy-6G– myeloid cells. After removing the column from the magnetic field, the magnetically retained Gr-1highLy-6G+ cells can be eluted as the positively selected cell fraction. To increase the purity, the positively selected cell fraction containing the Gr-1highLy-6G+ myeloid cells is separated over a second column. Gr-1dimLy-6G– myeloid cells that are contained in the first flow through fraction are indirectly magnetically labeled with Anti-Gr-1-Biotin (MDSC-Kit) and Streptavidin MicroBeads (MDSC-Kit) and isolated by positive selection from the pre-enriched Gr-1dimLy-6G– myeloid cell fraction. The magnetically labeled Gr-1dimLy-6G– myeloid cells are retained on the column and eluted after removal of the column from the magnetic field. To increase the purity, the positively selected cell fraction containing the Gr-1dimLy-6G– myeloid cells is separated over a second column.

1. Description

Splenocytes

Positive selection/ depletion of Gr-1highLy-6G+ cells

1. Indirect magnetic labeling of Gr-1highLy-6G+ cells with Anti-Ly-6G-Biotin and Anti-Biotin MicroBeads.
2. Magnetic separation using an LS Column (optional 2 LS Columns) or an autoMACS® Column (program "Posseld2").

Pre-enriched Gr-1dimLy-6G– cells (flow-through fraction) and enriched Gr-1highLy6G+ cells (positive selected fraction)

Positive selection of Gr-1dimLy-6G– cells

1. Indirect magnetic labeling of pre-enriched Gr-1dimLy-6G– with Anti-Gr-1-Biotin and Streptavidin MicroBeads (MDSC-Kit).
2. Magnetic separation using two MS Columns or autoMACS Columns (program "Posseld2").

Enriched Gr-1dimLy-6G– cells

1. Description

1.2 Background information

Myeloid-derived suppressor cells (MDSCs) are a heterogeneous population of immature myeloid cells with a remarkable capacity to induce T cell dysfunction. Phenotypically characterized as CD11b+Gr-1+–expressing cells, partial blocking of the cell differentiation of immature myeloid progenitor cells into granulocytes, macrophages, or dendritic cells results in the expansion of this population in various pathological conditions (cancer, infection, autoimmune diseases). Activation of MDSCs causes an increased production of immune suppressive factors Arginase 1 and iNOS, reactive oxygen species (ROS) and nitric oxide (NO) and thereby influences the T cell proliferation capacity, induces T cell apoptosis, or lead to unresponsiveness of T cells.[1–5]

The ability to suppress T cell function by MDSCs isolated with the Myeloid-Derived Suppressor Cell Isolation Kit was determined using CT26, 4T1, MCA203, and EL-4 tumor models in BALB/c and C57BL/6 mice, respectively.

The Myeloid-Derived Suppressor Cell Isolation Kit has been developed for the isolation of polymorphonuclear Gr-1highLy-6G+ and mononuclear Gr-1dimLy-6G– myeloid cells.[6] Gr-1dimLy-6G– cells can be further subdivided according to the expression of the marker Ly-6C into Gr-1dimLy-6ChighLy-6G– and Gr-1dimLy-6ClowLy-6G– cells. The proportion between Ly-6Chigh and Ly-6Clow subpopulations depends on the used tumor models.

1. Description

1.3 Applications

- Isolation of MDSCs from spleen and tumors.

  ▲ **Note:** The Myeloid-Derived Suppressor Cell Isolation Kit is not suitable for the isolation of MDSCs from bone marrow.

- Isolation of MDSCs for the analysis of their functional properties, e.g., on T cell activation and effector functions in different experimental mouse models.
- Isolation of MDSCs for phenotypical analysis.
- Isolation of MDSCs for downstream analysis such as gene expression profiling.

1.4 Reagent and instrument requirements

- Buffer: Prepare a solution containing phosphate-buffered saline (PBS), pH 7.2, 0.5% bovine serum albumin (BSA), and 2 mM EDTA by diluting MACS® BSA Stock Solution (# 130-091-376) 1:20 with autoMACS® Rinsing Solution (# 130-091-222). Keep buffer cold (2–8 °C). Degas buffer before use, as air bubbles could block the column.

  ▲ **Note:** EDTA can be replaced by other supplements such as anticoagulant citrate dextrose formula-A (ACD-A) or citrate phosphate dextrose (CPD). BSA can be replaced by other proteins such as mouse serum albumin, mouse serum, or fetal bovine serum (FBS). Buffers or media containing Ca^{2+} or Mg^{2+} are not recommended for use.

- MACS® Columns and MACS Separators: Positive selection of Gr-1^high Ly-6G⁺ cells can be performed by using two LS Columns. Gr-1^dim Ly-6G⁻ cells can be enriched by first depletion of Ly-6G⁺ cells by using an LS Column and subsequent positive selection of Gr-1⁺ cells by using two MS Columns. Positive selection or depletion can also be performed by using the autoMACS® Pro or the autoMACS Separator.

Column	Max. number of labeled cells	Max. number of total cells	Separator
**Positive selection**			
LS	10^8	2×10^9	MidiMACS, QuadroMACS, SuperMACS
**Second positive selection**			
MS	10^7	2×10^8	MiniMACS, OctoMACS, SuperMACS
**Positive selection**			
autoMACS	2×10^8	4×10^9	autoMACS Pro, autoMACS

▲ **Note:** Column adapters are required to insert certain columns into the SuperMACS™ Separator. For details see the respective MACS Separator data sheet.

- (Optional) Fluorochrome-conjugated antibodies for flow cytometric analysis, e.g., Anti-Ly-6C-FITC , Anti-Ly-6G-PE, Anti-Gr-1-PE, and CD11b-APC. For more information about antibodies refer to www.miltenyibiotec.com.
- (Optional) Propidium Iodide Solution (# 130-093-233) or 7-AAD Staining Solution (# 130-111-568) for flow cytometric exclusion of dead cells.
- (Optional) Dead Cell Removal Kit (# 130-090-101) for the depletion of dead cells.
- (Optional) Pre-Separation Filters (# 130-041-407) to remove cell clumps.

2. Protocol

2.1 Sample preparation

Prepare a single-cell suspension from spleen and tumors using a standard preparation method. For details see the protocols section at www.miltenyibiotec.com/protocols.

▲ The Myeloid-Derived Suppressor Cell Isolation Kit, mouse is not suitable for isolation of MDSCs from bone marrow.

▲ Dead cells may bind non-specifically to MACS MicroBeads. To remove dead cells, it is recommended using density gradient centrifugation or the Dead Cell Removal Kit (# 130-090-101).

2.2 Magnetic labeling of Gr-1^high Ly-6G⁺ cells

▲ Work fast, keep cells cold, and use pre-cooled solutions. This will prevent capping of antibodies on the cell surface and non-specific cell labeling.

▲ Volumes for magnetic labeling given below are for up to 10^8 total cells. When working with fewer than 10^8 cells, use the same volumes as indicated. When working with higher cell numbers, scale up all reagent volumes and total volumes accordingly (e.g. for 2×10^8 total cells, use twice the volume of all indicated reagent volumes and total volumes).

▲ For optimal performance it is important to obtain a single-cell suspension before magnetic labeling. Pass cells through 30 µm nylon mesh (Pre-Separation Filters, # 130-041-407) to remove cell clumps which may clog the column. Moisten filter with buffer before use.

▲ The recommended incubation temperature is 2–8 °C. Working on ice may require increased incubation times. Higher temperatures and/or longer incubation times may lead to non-specific cell labeling.

1. Determine cell number.
2. Centrifuge cell suspension at 300×g for 10 minutes at 4 °C. Aspirate supernatant completely.
3. Resuspend cell pellet in 350 µL of buffer per 10^8 total cells.
4. Add 50 µL of FcR Blocking Reagent per 10^8 total cells.
5. Mix well and incubate for 10 minutes in the refrigerator (2–8 °C).
6. Add 100 µL of Anti-Ly-6G-Biotin (MDSC-Kit).
7. Mix well and incubate for 10 minutes in the refrigerator (2–8 °C).
8. Wash cells by adding 5–10 mL of buffer per 10^8 cells and centrifuge at 300×g for 10 minutes at 4 °C. Aspirate supernatant completely.
9. Resuspend up to 10^8 cells in 800 µL of buffer.
10. Add 200 µL of Anti-Biotin MicroBeads.
11. Mix well and incubate for 15 minutes in the refrigerator (2–8 °C).
12. Wash cells by adding 10–20 mL of buffer per 10^8 cells and centrifuge at 300×g for 10 minutes at 4 °C. Aspirate supernatant completely.
13. Resuspend up to 10^8 cells in 500 µL of buffer.
    ▲ **Note:** For higher cell numbers, scale up buffer volume accordingly.
14. Proceed to magnetic separation (2.3).

2. Protocol

2.3 Magnetic separation: Positive selection of Gr-1^high Ly-6G⁺ cells

▲ Choose an appropriate MACS® Column and MACS Separator according to the number of total cells and the number of Gr-1^high Ly-6G⁺ cells. For details see table in section 1.4.

▲ Always wait until the column reservoir is empty before proceeding to the next step.

Magnetic separation with LS Columns

1. Place LS Column in the magnetic field of a suitable MACS Separator. For details see the respective MACS Column data sheet.

2. Prepare column by rinsing with 3 mL of buffer.
3. Apply cell suspension onto the column. Collect flow-through containing unlabeled cells.
4. Wash column with 3×3 mL of buffer. Collect unlabeled cells that pass through and combine with the effluent from step 3; this is the unlabeled pre-enriched $Gr\text{-}1^{dim}Ly\text{-}6G^{-}$ cell fraction.

   ▲ **Note:** Perform washing steps by adding buffer aliquots only when the column reservoir is empty.
5. Remove column from the separator and place it on a suitable collection tube.
6. Pipette 5 mL of buffer onto the column. Immediately flush out the magnetically labeled cells by firmly pushing the plunger into the column.
7. To increase the purity of $Gr\text{-}1^{high}Ly\text{-}6G^{+}$ cells, the eluted fraction should be enriched over a second LS Column. Repeat the magnetic separation procedure as described in steps 1 to 6 by using a new column.
8. Proceed to 2.4 for further isolation of $Gr\text{-}1^{dim}Ly\text{-}6G^{-}$ cells.

Magnetic separation with the autoMACS® Pro Separator or the autoMACS Separator

▲ Refer to the respective user manual for instructions on how to use the autoMACS Pro Separator or the autoMACS Separator.

▲ Buffers used for operating the autoMACS Pro Separator or the autoMACS Separator should have a temperature of ≥ 10 °C.

▲ Program choice depends on the isolation strategy, the strength of magnetic labeling, and the frequency of magnetically labeled cells. For details refer to the section describing the cell separation programs in the respective user manual.

Magnetic separation with the autoMACS Pro Separator

1. Prepare and prime the instrument.
2. Apply tube containing the sample and provide tubes for collecting the labeled and unlabeled cell fractions. Place sample tube in row A of the tube rack and the fraction collection tubes in rows B and C.
3. For a standard separation choose the program: "Posseld2"

   Collect negative fraction in row B of the tube rack. This fraction represents the unlabeled and pre-enriched $Gr\text{-}1^{dim}Ly\text{-}6G^{-}$ cells.

   Collect positive fraction in row C of the tube rack. This fraction represents the magnetically labeled $Gr\text{-}1^{high}$ $Ly\text{-}6G^{+}$ cells.

Magnetic separation with the autoMACS® Separator

1. Prepare and prime the instrument.
2. Apply tube containing the sample and provide tubes for collecting the labeled and unlabeled cell fractions. Place sample tube at the uptake port and the fraction collection tubes at port neg1 and port pos2.
3. For a standard separation choose the program: "Posseld2"

   Collect negative fraction from outlet port neg1. This fraction represents the unlabeled and pre-enriched $Gr\text{-}1^{dim}Ly\text{-}6G^{-}$ cells.

   Collect positive fraction outlet port pos2. This fraction represents the magnetically labeled $Gr\text{-}1^{high}$ $Ly\text{-}6G^{+}$ cells.

2.4 Magnetic labeling of $Gr\text{-}1^{dim}Ly\text{-}6G^{-}$ cells

▲ Volumes for magnetic labeling given below are for an initial starting cell number of up to 10^8 total cells. When working with fewer than 10^8 cells, use the same volumes as indicated. When working with higher cell numbers, scale up all reagent volumes and total volumes accordingly (e.g. for 2×10^8 total cells, use twice the volume of all indicated reagent volumes and total volumes).

1. Centrifuge cell suspension at 300×g for 10 minutes at 4 °C. Aspirate supernatant completely.
2. Resuspend cell pellet in 400 µL of buffer per 10^8 total cells.
3. Add 100 µL of Anti-Gr-1-Biotin (MDSC-Kit) per 10^8 total cells.
4. Mix well and incubate for 10 minutes in the refrigerator (2–8 °C).
5. Wash cells by adding 5–10 mL of buffer per 10^8 cells and centrifuge at 300×g for 10 minutes at 4 °C. Aspirate supernatant completely.
6. Resuspend cell pellet in 900 µL of buffer per 10^8 total cells.
7. Add 100 µL of Streptavidin MicroBeads (MDSC-Kit).
8. Mix well and incubate for 15 minutes in the refrigerator (2–8 °C).
9. Wash cells by adding 10–20 mL of buffer per 10^8 cells and centrifuge at 300×g for 10 minutes at 4 °C. Aspirate supernatant completely.
10. Resuspend up to 10^8 cells in 500 µL of buffer.

    ▲ **Note:** For higher cell numbers, scale up buffer volume accordingly.
11. Proceed to magnetic separation (2.5).

2.5 Magnetic separation: Positive selection of $Gr\text{-}1^{dim}Ly6G^{-}$ cells

▲ To achieve highest purities, perform two consecutive column runs.

1. Place MS Column in the magnetic field of a suitable MACS® Separator. For details see MS Column data sheet.
2. Prepare column by rinsing with 500 µL of buffer.
3. Apply cell suspension onto the column.

4. Collect unlabeled cells that pass through and wash column with 3×500 µL of buffer. Collect total effluent; this is the unlabeled cell fraction.

   ▲ **Note:** Perform washing steps by adding buffer aliquots only when the column reservoir is empty.

5. Remove column from the separator and place it on a suitable collection tube.

   ▲ **Note:** To perform a second column run, cells may be eluted directly from the first onto the second, equilibrated column instead of a collection tube.

6. Pipette 1 mL of buffer onto the column. Immediately flush out the magnetically labeled cells (Gr-1^{dim}Ly-$6G^{-}$) by firmly pushing the plunger into the column.

7. To increase purity of Gr-1^{dim}Ly-$6G^{-}$ cells, enrich the eluted fraction over a second MS Column. Repeat the magnetic separation procedure as described in steps 1 to 6 by using a new column.

Magnetic separation with the autoMACS® Pro Separator or the autoMACS Separator

▲ Refer to the respective user manual for instructions on how to use the autoMACS Pro Separator or the autoMACS Separator.

▲ Buffers used for operating the autoMACS Pro Separator or the autoMACS Separator should have a temperature of ≥ 10 °C.

▲ Program choice depends on the isolation strategy, the strength of magnetic labeling, and the frequency of magnetically labeled cells. For details refer to the section describing the cell separation programs in the respective user manual.

Magnetic separation with the autoMACS Pro Separator

1. Prepare and prime the instrument.
2. Apply tube containing the sample and provide tubes for collecting the labeled and unlabeled cell fractions. Place sample tube in row A of the tube rack and the fraction collection tubes in rows B and C.
3. For a standard separation choose the program: "Posseld2"

   Collect positive fraction in row C of the tube rack. This fraction represents the magnetically labeled Gr-1^{dim}Ly-$6G^{-}$ cells.

Magnetic separation with the autoMACS Separator

1. Prepare and prime the instrument.
2. Apply tube containing the sample and provide tubes for collecting the labeled and unlabeled cell fractions. Place sample tube at the uptake port and the fraction collection tubes at port neg1 and port pos2.
3. For a standard separation choose the program: "Posseld2"

   Collect positive fraction from outlet port pos2. This fraction represents the magnetically labeled Gr-1^{dim}Ly-$6G^{-}$ cells.

3. Example of a separation using the Myeloid-Derived Suppressor Cell Isolation Kit

Myeloid-derived suppressor cells were isolated from a spleen of a BALB/c mouse using the Myeloid-Derived Suppressor Cell Isolation Kit, two LS Columns, two MS Columns, and an appropriate MACS® Separator. Cells were fluorescently stained with Anti-Ly-6C-FITC, CD11b-APC, and Anti-Gr-1-PE or Anti-Ly-6G-PE, respectively, and analyzed by flow cytometry. Cell debris and dead cells were excluded from the analysis based on scatter signals and propidium iodide fluorescence.

Spleen cells before separation

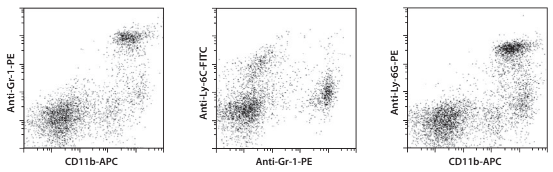

Enriched Gr-1^{high}Ly-$6G^{+}$ cells

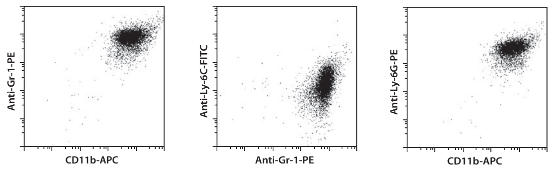

Pre-enriched Gr-1^{dim}Ly-$6G^{-}$ cells

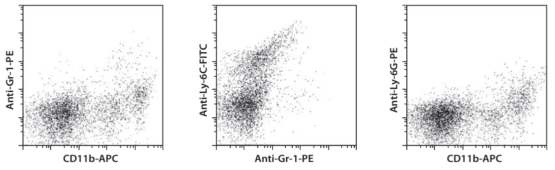

Enriched Gr-1^{dim}Ly-$6G^{-}$ cells

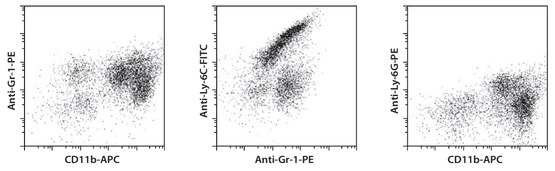

4. References

1. Serafini, P. *et al.* (2004) Derangement of immune responses by myeloid suppressor cells. Cancer Immunol Immunother 53: 64–72.
2. Youn, J. *et al.* (2008) Subsets of Myeloid-Derived Suppressor Cells in Tumor-Bearing Mice. J. Immunol. 181: 5791–5802.
3. Ostrand-Rosenberg, S. and Sinha, P. (2009) Myeloid-Derived Suppressor Cells: Linking Inflammation and Cancer. J. Immunol. 182: 4499–4506.
4. Gabrilovich, D. I. and Nagaraj, S. (2009) Myeloid-derived suppressor cells as regulators of the immune system. Nat. Rev. Immunol. 9: 162–174.
5. Bronte, V. *et al.* (1999) Unopposed production of granulocyte-macrophage colony-stimulating factor by tumors inhibits $CD8^+$ T cell responses by dysregulating antigen-presenting cell maturation. J. Immunol. 162: 5728–5737.
6. Movahedi, K. *et al* (2008) Identification of discrete tumor-induced myeloid-derived subpopulations with distinct T cell suppressive activity. Blood 111: 4233–4244.

Refer to **www.miltenyibiotec.com** for all data sheets and protocols. Miltenyi Biotec provides technical support worldwide. Visit www.miltenyibiotec.com/local to find your nearest Miltenyi Biotec contact.

Legal notices

Limited product warranty

Miltenyi Biotec B.V. & Co. KG and/or its affiliate(s) warrant this product to be free from material defects in workmanship and materials and to conform substantially with Miltenyi Biotec's published specifications for the product at the time of order, under normal use and conditions in accordance with its applicable documentation, for a period beginning on the date of delivery of the product by Miltenyi Biotec or its authorized distributor and ending on the expiration date of the product's applicable shelf life stated on the product label, packaging or documentation (as applicable) or, in the absence thereof, ONE (1) YEAR from date of delivery ("Product Warranty"). Miltenyi Biotec's Product Warranty is provided subject to the warranty terms as set forth in Miltenyi Biotec's General Terms and Conditions for the Sale of Products and Services available on Miltenyi Biotec's website at www.miltenyibiotec.com, as in effect at the time of order ("Product Warranty"). Additional terms may apply. BY USE OF THIS PRODUCT, THE CUSTOMER AGREES TO BE BOUND BY THESE TERMS.

THE CUSTOMER IS SOLELY RESPONSIBLE FOR DETERMINING IF A PRODUCT IS SUITABLE FOR CUSTOMER'S PARTICULAR PURPOSE AND APPLICATION METHODS.

Technical information

The technical information, data, protocols, and other statements provided by Miltenyi Biotec in this document are based on information, tests, or experience which Miltenyi Biotec believes to be reliable, but the accuracy or completeness of such information is not guaranteed. Such technical information and data are intended for persons with knowledge and technical skills sufficient to assess and apply their own informed judgment to the information. Miltenyi Biotec shall not be liable for any technical or editorial errors or omissions contained herein.

All information and specifications are subject to change without prior notice. Please contact Miltenyi Biotec Technical Support or visit www.miltenyibiotec.com for the most up-to-date information on Miltenyi Biotec products.

Licenses

This product and/or its use may be covered by one or more pending or issued patents and/or may have certain limitations. Certain uses may be excluded by separate terms and conditions. Please contact your local Miltenyi Biotec representative or visit Miltenyi Biotec's website at www.miltenyibiotec.com for more information.

The purchase of this product conveys to the customer the non-transferable right to use the purchased amount of the product in research conducted by the customer (whether the customer is an academic or for-profit entity). This product may not be further sold. Additional terms and conditions (including the terms of a Limited Use Label License) may apply.

CUSTOMER'S USE OF THIS PRODUCT MAY REQUIRE ADDITIONAL LICENSES DEPENDING ON THE SPECIFIC APPLICATION. THE CUSTOMER IS SOLELY RESPONSIBLE FOR DETERMINING FOR ITSELF WHETHER IT HAS ALL APPROPRIATE LICENSES IN PLACE. Miltenyi Biotec provides no warranty that customer's use of this product does not and will not infringe intellectual property rights owned by a third party. BY USE OF THIS PRODUCT, THE CUSTOMER AGREES TO BE BOUND BY THESE TERMS.

Trademarks

autoMACS, MACS, MidiMACS, the Miltenyi Biotec logo, MiniMACS, OctoMACS, QuadroMACS, and SuperMACS are registered trademarks or trademarks of Miltenyi Biotec and/or its affiliates in various countries worldwide.

Copyright © 2020 Miltenyi Biotec and/or its affiliates. All rights reserved.
